# Incidence and aetiology of diarrhoeal disease among children under five years in the post-rotavirus vaccine introduction era in Manhiça District, Mozambique

**DOI:** 10.1186/s41182-026-01095-2

**Published:** 2026-10-19

**Authors:** Percina Chirinda, Llorenç Quintó, Corssino Tchavana, Augusto Messa, Arsénio Nhacolo, Sozinho Acacio, Tacilta Nhampossa, Quique Bassat, Goitom G. Weldegebriel, Jacqueline E. Tate, Umesh Parashar, Jason M. Mwenda, Filomena Manjate, Inacio Mandomando

**Affiliations:** 1 Centro de Investigação em Saúde de Manhiça (CISM), 1929 Maputo, Mozambique; 2 ISGlobal, 08036 Barcelona, Spain; 3 Facultat de Medicina i Ciències de la Salut, Universitat de Barcelona (UB), 08036 Barcelona, Spain; 4 Instituto Nacional de Saúde (INS), 1120 Marracuene, Maputo Mozambique; 5 ICREA, Pg. Lluís Companys 23, 08010 Barcelona, Spain; 6 Institut Clínic de Medicina I Dermatologia, Hospital Clínic de Barcelona, Barcelona, Spain; 7 Pediatrics Department, Hospital Sant Joan de Déu, Universitat de Barcelona, Esplugues, Barcelona, Spain; 8 CIBER de Epidemiología y Salud Pública, Instituto de Salud Carlos III, Madrid, Spain; 9 World Health Organization, Intercountry Support Team, East and Southern Africa, Harare, Zimbabwe; 10 Centers for Disease Control and Prevention (CDC), Atlanta, GA 30333 USA; 11 Diarrhea Pathogens Research Unit, Department of Virology, Sefako Makgatho University of Pretoria, Pretoria, South Africa; 12 Kenya Primate Research Institute, Nairobi, Kenya; 13 Global Health and Tropical Medicine, GHTM, Associate Laboratory in Translation and Innovation Towards Global Health, LA-REAL, Instituto de Higiene e Medicina Tropical, IHMT, Universidade NOVA de Lisboa, UNL, Lisbon, Portugal

**Keywords:** Diarrhoea, Incidence, Aetiology, Vaccine, Mozambique

## Abstract

**Background:**

Diarrhoea remains a major cause of death in children under five, despite the global decline in hospitalisations following the widespread introduction of rotavirus vaccines . As rotavirus-associated disease declines, it is important to identify other diarrhoea associated pathogens, although post-vaccine data remain limited in Mozambique. This study aimed to estimate the incidence and determine the aetiology of Moderate-to-Severe Diarrhoea (MSD) and Less Severe Diarrhoea (LSD) among children under five, post-rotavirus vaccine introduction in Manhiça District, a rural setting in Mozambique.

**Methods:**

We estimated the incidence of diarrhoea using data from the morbidity surveillance and pathogen-attributable fractions based on analysis of stool specimens collected in a case–control study.

**Results:**

The overall incidence of MSD was 5.6 cases per 1,000 child-years at risk (CYAR), with the highest rate observed among infants aged 0–11 months (15.5 per 1,000 CYAR) with a declining trend over time. LSD incidence was approximately 20 times higher than MSD incidence (119.7 per 1,000 CYAR), peaking among children aged 12–23 months (232.9 per 1,000 CYAR). Rotavirus remained the leading cause of both MSD and LSD among infants aged 0–11 months, while other important pathogens associated with LSD in this age group included sapovirus and *Cryptosporidium* spp. Among children aged 12–23 months, enteroaggregative *Escherichia coli* and adenovirus 40/41 were the major contributors to MSD.

**Conclusions:**

Our findings highlight that rotavirus remains an important diarrhoea aetiology among infants and demonstrate changes on diarrhoea aetiologies post-rotavirus vaccine introduction; reinforcing the need for continued surveillance to better inform control and prevention strategies.

**Supplementary Information:**

The online version contains supplementary material available at https://doi.org/10.1186/s41182-026-01095-2.

## Background

Despite global decline in diarrhoea-related hospitalisations and mortality, diarrhoea remains among the leading causes of death in children under five years of age. This decline has been largely attributed to the widespread introduction of rotavirus vaccines and gradual but significant improvements in Water, Sanitation, and Hygiene (WASH) conditions [1, 2]. Globally, diarrhoea accounts for 443,832 deaths annually [3], with the highest burden in Low- and Middle-income Countries (LMICs) [4]. Mozambique, introduced the monovalent rotavirus vaccine (Rotarix®, GlaxoSmithKline Biologicals, Rixensart, Belgium) into the country expanded immunization program in September 2015, following The World Health Organization (WHO) recommendations for rotavirus vaccine adoption in countries with high burden of disease [5]. This introduction was supported by findings from the Global Enteric Multicenter Study (GEMS), which demonstrated that rotavirus was the leading cause of Moderate-to-Severe Diarrhoea (MSD) and Less-Severe Diarrhoea (LSD), with attributable fractions (AFs) of 35% and 20%, respectively [6, 7]. Following rotavirus vaccine introduction, the *Centro de Investigação em Saúde de Manhiça* (CISM) established a diarrhoeal disease surveillance in Manhiça District, to monitor the vaccine impact and the epidemiology of other enteric pathogens. Among this surveillance, we reported a significant reduction in all-cause diarrhoea and laboratory-confirmed rotavirus infection post-vaccine introduction [8]. The decline of rotavirus-associated diarrhoea triggered interest to identify other enteric pathogens contributing to disease burden. Several studies have been conducted in various settings to characterise childhood diarrhoea aetiologies to inform policy and prioritize interventions. Despite methodological differences, these studies consistently identify rotavirus as the leading cause of MSD post-vaccine introduction. Other important aetiologies associated with diarrhoea hospitalisations among children under five included *Shigella*, *Cryptosporidium*, heat-stable enterotoxin–producing enterotoxigenic *Escherichia coli* (ST-ETEC), adenovirus, and norovirus [9–11]. Available studies in Mozambique, limited their methodology on rotavirus characterization, although there are reports of enteric parasites and other viral pathogens [12–15]. To date, no comprehensive post-vaccine assessment has been conducted to evaluate the broader epidemiologic and aetiological landscape of diarrhoeal disease in the country. This study estimated the incidence and the aetiology of MSD and LSD among children aged 0–59 months in Manhiça District post-rotavirus vaccine introduction (2015–2021).

## Methodology

### Study area

This study was conducted in Manhiça, a rural district located 80 km north of Mozambique’s capital, Maputo City. Manhiça has a subtropical climate, with a warm, rainy season from November to April and a cooler, dry season during the rest of the year. The geographical and socio-demographic characteristics of the Manhiça District community have been previously described [16]. CISM established a continuous Health and Demographic Surveillance System (HDSS) in the district in 1996, covering about 201,845 inhabitants [17] to routinely collect demographic data. The HDSS is linked to a 24-h hospital-based morbidity surveillance for children < 15 years old in seven health facilities of the district, where clinical data of outpatient and inpatient visits are routinely collected using standard case report forms.

### Study design and sample collection

This study was conducted in two components: the first component was based on analysis using the morbidity surveillance data to estimate the incidence of acute diarrhoeal episodes among children aged 0–59 months residing in the CISM’s HDSS area [16] from September 2015 to December 2021. During this period, all children aged 0–59 months under the morbidity surveillance were considered in the population at risk. Diarrhoea was defined as the occurrence of three or more loose or watery stools within 24 h. The diarrhoea cases were classified as MSD, defined as diarrhoea requiring hospitalization and accompanied by at least one of the following criteria: sunken eyes, loss of skin turgor; dysentery or administration of intravenous hydration [8], or LSD, defined as diarrhoea cases that did not meet the criteria for MSD [7].

The second component included analysis of data from a case–control study within the diarrhoeal disease surveillance, to estimate the pathogen-specific attributable fractions for MSD and LSD. The case–control study, was a passive surveillance conducted in three health facilities (Manhiça district Hospital, Xinavane Rural Hospital and Maragra Health Center) of Manhiça district. The detailed methodology of this study has been described [8]. Briefly, the study staff visited the outpatient and inpatient departments to identify children meeting the study eligibility criteria. Children aged 0–59 months registered within the morbidity surveillance were enrolled to the study if presenting with MSD or LSD after the parents or guardians have signed the informed consent. For each case, one to three healthy community controls were enrolled matched by age, sex, and neighbourhood. Recruitment into the case–control study did not cover the entire incidence observation period. MSD cases were enrolled from September 2015 to December 2021, whereas LSD cases and matched community controls were enrolled from April 2017 to December 2021.

Each case and control enrolled provided a fresh stool sample in a polyethylene container, which was refrigerated in a cool box with a cooler block (2–8◦C) until delivery to the laboratory. Additionally, we collected two faecal swabs from each sample, one in Cary-Blair medium and another in buffered glycerol saline (BGS), for bacterial culture.

### Laboratory procedures

We used commercial enzyme-linked immunosorbent assays (ELISA) to detect rotavirus (Prospect® Rotavirus, Oxoid, Ltd., Hampshire, UK); adenovirus (Prospect® Adenovirus, Oxoid, Ltd., Hampshire, UK); adenovirus 40/41 (Premier Adenoclone, Meridian Bioscience, Cincinnati, OH, USA); *Giardia duodenalis* (Giardia II, TechLab, Blacksburg, VA, USA); *Entamoeba histolytica* (E. Histolytica II, TechLab, Blacksburg, VA, USA) and *Cryptosporidium* spp. (Cryptosporidium II, TechLab, Blacksburg, VA, USA). Norovirus, astrovirus, and sapovirus were detected using a multiplex PCR with specific primers (Table S1) as previously described [18]. Diarrhoeal-associated bacteria of interest were identified and isolated using conventional bacterial culture as described [18]. Pathotypes of diarrhoeagenic *Escherichia coli* were assessed using multiplex PCR as described earlier [18], to identify enterotoxigenic *E. coli* (ETEC), typical and atypical enteropathogenic *E. coli* (EPEC), and enteroaggregative *E. coli* (EAEC) (used primers in Table S2).

### Data management and analysis

All the analyses were conducted using Stata version 14.1 (StataCorp LP, College Station, TX, USA) and R version 4.3.1 (R Core Team, 2023). We estimated minimum community incidence rates using data from passive surveillance of diarrhoea episodes detected at seven Manhiça healthcare facilities, registered through the morbidity surveillance. Time at risk was calculated in person-years. For each child, the start of follow-up was defined as the beginning of the study period (September 14, 2015) or birth (if born after September 14, 2015). Follow-up ended on the earliest of the following dates: December 31, 2021; the child’s date of death; or their fifth birthday. To avoid overestimation due to recurrent care seeking for the same illness, a 15-day period following each diarrhoea episode was excluded from the time at risk, as this period was considered the post-episode immunity period. For MSD, the number of hospitalizations days was also subtracted from the time at risk. The incidence rates were stratified into three age groups, 0–11, 12–23, and 24–59 months.

For the case–control component, pathogen-specific AFs were estimated separately for MSD and LSD, and within the age strata of 0–11, 12–23, and 24–59 months. For each pathogen, we fitted an unconditional logistic regression model with case–control status as the dependent variable and detection of the corresponding pathogen as the independent variable. These were crude pathogen-specific models and no additional covariates were included. The AF and its 95% confidence interval were subsequently estimated from the fitted logistic model using the model-based method described by Greenland and Drescher [19], as implemented with the punafcc command in Stata [35]. Missing pathogen results were not imputed; each analysis included participants with an available laboratory result for the pathogen under evaluation, and therefore the effective sample size varied by pathogen. Co-detections were retained, such that participants with multiple pathogens could contribute to more than one pathogen-specific analysis. Although controls were selected according to the original study matching criteria, the AF analysis did not incorporate the matched sets through conditional logistic regression. MSD recruitment started earlier than recruitment of LSD cases and community controls; however calendar period was not explicitly included in the AF models. Chi-square or Fisher’s exact tests were applied, when appropriate, to compare categorical variables, with a significance level of 5%. Baseline characteristics were summarized descriptively with no significance testing performed, as these characteristics were balanced by design.

## Results

### Incidence of diarrhoea post-rotavirus vaccine introduction

**Fig. 1:**
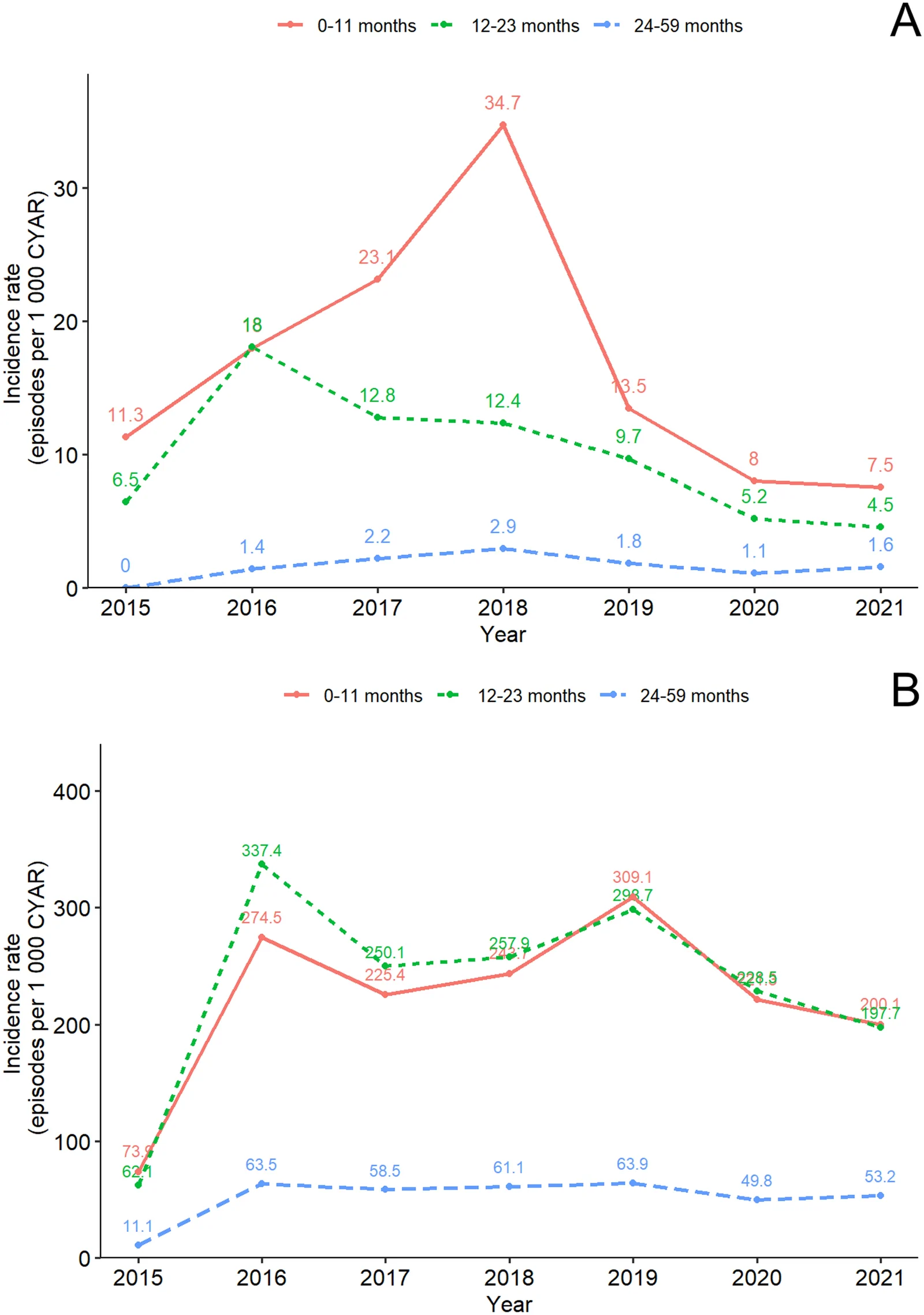
Yearly community incidence rates of diarrhoea in children aged 0–59 months in Manhiça district from September 2015 to December 2021. **A** Moderate to severe (MSD). **B** Less severe diarrhoea (LSD). Data for 2015 include only four months of surveillance (September–December) and should be interpreted with caution, as they may not fully reflect seasonal patterns

During the study period (September 14, 2015–December 31, 2021), children contributed to a total of 107,334.46 child-years at risk (CYAR) for MSD and 111,330.92 CYAR for LSD. From the 13,936 episodes observed during this period, 606 were classified as MSD, yielding an overall minimum community incidence rate (MCIR) of 5.6 cases per 1,000 CYAR (Table 1). We observed an initial increase in the annual MCIR within the 0–11 months age group during the first years of the study, followed by a decline from 2018–2019, which continued through 2021 (Fig. 1A). A similar pattern was observed among children aged 12–23 months, with incidence increasing from 2015–2016 and declining from 2016 to 2021. The overall yearly incidence among 24–59 months age group, increased from 2015–2018, followed by a decline through 2020 and a slight increase in 2021 (Fig. 1A).

**Table 1:** Minimum community incidence rates of Moderate to severe Diarrhoea and Less severe Diarrhoea by age group in Manhiça District, Mozambique, September 2015–December 2021

Age groups	Episodes	CYAR	Incidence rate per 1000 CYAR (95% CI)	Incidence rate ratio (95% CI)	*p* value
Moderate to Severe Diarrhoea (MSD)					
0–11 months^a^	298	19,214.05	15.5 (13.8–17.4)	–	
12–23 months	201	20,322.51	9.9 (8.6–11.4)	0.64 (0.53–0.76)	0.001
24–59 months	107	67,797.90	1.6 (1.3–1.9)	0.10 (0.08–0.12)	0.001
All ages	606	107,334.46	5.6 (5.2–6.1)	–	
Less Severe Diarrhoea (LSD)					
0–11 months^a^	4,793	21,626.45	221.6 (215.4–228)	–	
12–23 months	5,044	21,653.62	232.9 (226.6–239.5)	1.05 (1.01–1.09)	0.050
24–59 months	3,493	68,050.85	51.3 (49.6–53.1)	0.232 (0.222–0.242)	0.001
All ages	13,330	111,330.92	119.7 (117.7–121.8)	–	

CYAR: Child-years at risk

^a^0–11 months was the reference group for incidence rate ratio calculation

On the other hand, 13,330 episodes were classified as LSD, yielding a minimum incidence rate of 119.7 cases per 1,000 CYAR. The 12–23 months group had the highest MCIR (232.9 cases per 1,000 CYAR), followed by the 0–11 months group (Incidence Rate Ratio [IRR] = 1.05; *p* = 0.05) (Table 1). In general, LSD annual incidence rates varied across the study period in all age groups, with peaks in 2016 and 2019. Following the 2019 peak, the incidence declined in 2020 among all age groups. This decline continued to 2021 in children aged 0–11 and 12–23 months. However, a slight increase was observed among children aged 24–59 months (Fig. 1B).

### Case control component

#### General characteristics and clinical presentation

Between September 2015 and December 2021, we enrolled 1,907 participants in the case control component, comprising 807 cases and 1,100 controls. Among the cases, 41% (n = 334) presented with MSD, and 59% (n = 473) had LSD. Nearly half of the cases were children aged 0–11 months (49%), followed by 12–23 months (33%). About 57% of cases were male, 80% had received rotavirus vaccine and 74% were HIV-negative. Most cases experienced watery diarrhoea (79%), lasting 1–4 days (92%), while 4–5 episodes/24 h were reported in 47% of cases. Vomiting was present in 41% of the cases, fever in 38%, dehydration in 98%, and stunting in 27% (Table 2).

**Table 2:** Demographic and clinical characteristics of cases with diarrhoea and their respective controls enrolled in the Manhiça District, between 2015 and 2021

Characteristics	Cases N = 807 n (%)	Controls N = 1100 n (%)	Total N = 1907
Study case			
MSD	334 (41)	312 (28)	–
LSD	473 (59)	788 (72)	–
Age group			
0–11 months	397 (49)	430 (39)	827
12–23 months	265 (33)	444 (40)	709
24–59 months	145 (18)	226 (18)	371
Sex			
Male	458 (57)	596 (54)	1054
Female	349 (43)	504 (45)	853
Rotavirus vaccination status			
Vaccinated	644 (80)	860 (78)	1504
Unvaccinated	127 (16)	194 (18)	321
Missing	36 (4)	46 (4)	82
HIV status			
Positive	44 (5)	108 (10)	152
Negative	600 (74)	795 (72)	1395
Missing	163 (20)	197 (18)	360
Diarrhoea duration			
1–4 days	731 (92)	NA	792
5 days	22 (3)		
> 6 days	39 (5)		
Diarrhoea episodes/24 h			
1 to 3	125 (16)	NA	782
4 to 5	369 (47)		
≥ 6	288 (37)		
Stool characteristics			
Watery	606 (79)		
Pasty	138 (18)	NA	
Mucous	17 (2)		762
Bloody	6 (1)		
Vomit			
Yes	330 (41)	NA	800
Vomit duration			
1 day	202 (62)	NA	327
2 days	56 (17)		
≥ 3 days	69 (21)		
Fever			
Yes	304 (62)	NA	803
Dehydration^a^			
Yes	327 (59)	NA	332
Malnutrition			
Stunting	186 (27)	NA	686
Wasting	140 (19)	NA	702
Underweight	151 (19)	NA	766

MSD: Moderate to Severe Diarrhoea; LSD: Less Severe Diarrhoea; NA: Not Applicable

^a^Dehydration status applicable for MSD cases only

#### Diarrhoea aetiology

Overall, the most detected pathogens were *G. duodenalis* (28%), enteroaggregative *Escherichia coli* (EAEC) (18%), rotavirus (17%), and *E. histolytica* (11%), whereas atypical enteropathogenic *E. coli* (1%), adenovirus 40/41 (2%), and sapovirus (3%) were the least frequently detected (Table S3).

**Fig. 2:**
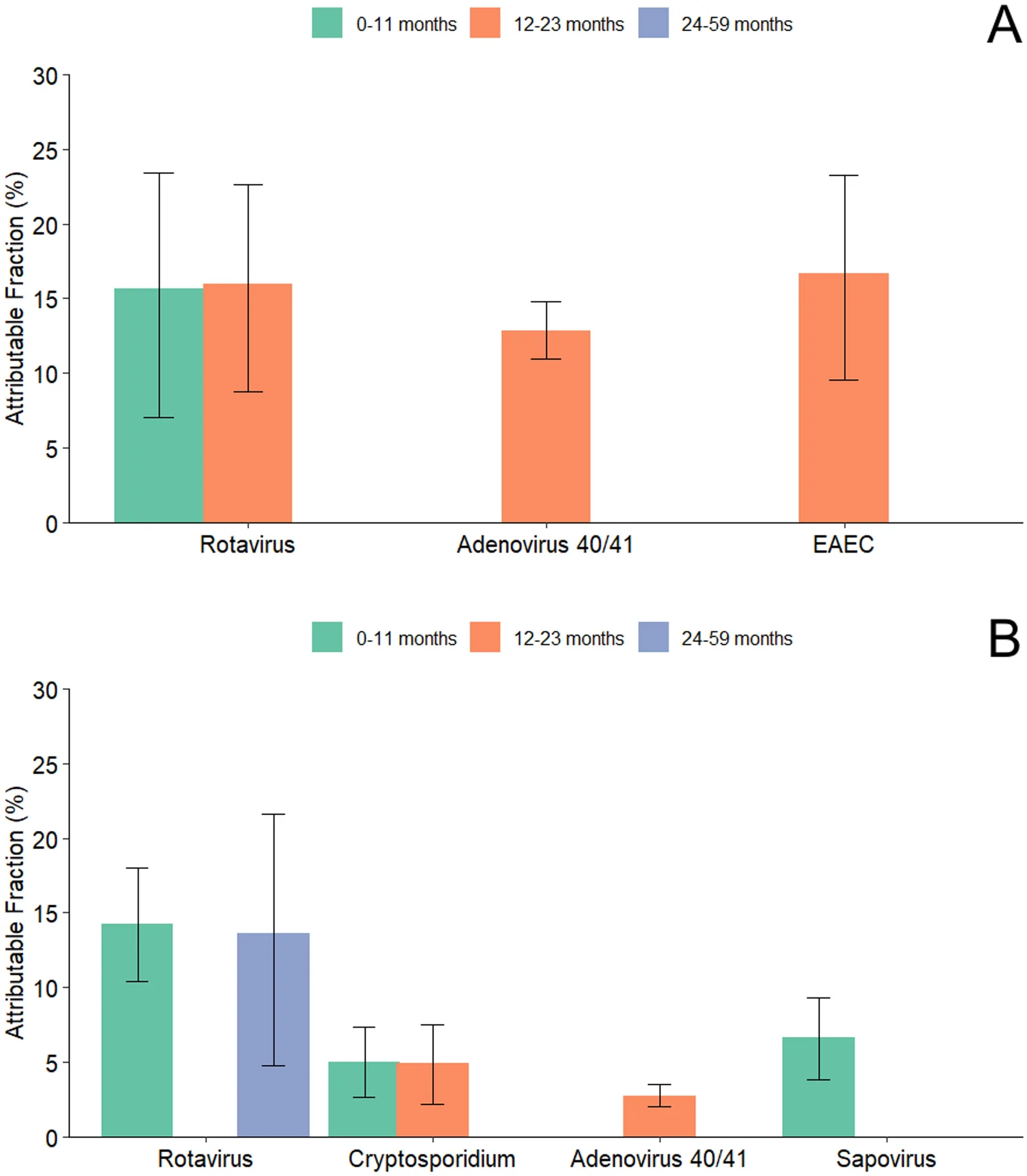
Pathogen-specific attributable fractions of pathogens associated with Diarrhoea stratified by age group in children under five years in Manhiça district Mozambique, September 2015–December 2021. **A** Moderate to severe diarrhoea (MSD). **B** Less severe diarrhoea (LSD)

Among children aged 0–11 months with MSD, rotavirus (28%) and EAEC (27%) were the most frequently detected pathogen, followed by *E. histolytica* (12%) (Table S3). Rotavirus was the only pathogen significantly associated with disease in this age group, with an attributable fraction (AF) of 15.6% (95% Confidence Interval [CI]: 6.9–23.4). In the 12–23 months age group, rotavirus (26%), EAEC (24%) and *G. duodenalis* (23%) were the most frequently detected pathogens (Table S3); EAEC exhibited the highest AF of 16.7% (95% CI 9.5–23.2), followed by rotavirus (15.9%; 95% CI 8.7–22.7) and adenovirus 40/41 (12.9%; 95% CI 10.9–14.7) (Fig. 2A). In the 24–59 months age group, *G. duodenalis* (29%), rotavirus (25%), and EAEC (18%) were the most frequently detected pathogens (Table S3), although none were significantly associated with MSD.

EAEC (26%), rotavirus (21%) and norovirus (12%) were the most frequent pathogens detected in LSD cases aged 0–11 months (Table S3). Rotavirus was the leading pathogen associated with disease, with an AF of 14.3% (95% CI 10.4–18.0), followed by sapovirus (AF = 6.6%; 95% CI 3.8–9.3) and *Cryptosporidium* spp. (AF = 5.0%; 95% CI 2.6–7.3). Among children aged 12–23 months, *G. duodenalis* (37%), rotavirus (20%) and *E. histolytica* (17%) were the most frequent detected pathogens (Table S3), yet *Cryptosporidium* spp. demonstrated the highest AF (4.9%; 95% CI 2.2–7.5), followed by adenovirus (AF = 2.8%; 95% CI 2.0–3.5). In the 24–59 months age group, *G. duodenalis* (44%), rotavirus (26%), and norovirus (15%) (Table S3) were most frequently detected, with rotavirus being the only pathogen significantly associated with LSD (AF = 13.6%; 95% CI 4.7–21.6) (Fig. 2B).

## Discussion

This study estimated the minimum community incidence as well as the aetiology and AF of different pathogens causing MSD and LSD in children aged 0–59 months in Manhiça district, southern Mozambique. We documented a substantial reduction in the incidence of MSD post-rotavirus vaccine introduction, although rotavirus remained the leading pathogen associated with diarrhoea. Children aged 0–11 months had significantly higher incidence rates, compared to older children, consistent with available literature indicating greater susceptibility among infants [19]. Age-specific trend analysis of MSD incidence rate showed higher incidences among children aged 0–11 months in the first 2/3 years post vaccine introduction, followed by a significant decline. This temporal pattern coincided with the gradual scale-up of rotavirus vaccination in Mozambique, as indicated by the increase in vaccine coverage from 17% in 2015 to 65% in 2016 and 79% in 2017 [1]. Although vaccine coverage has generally remained high nationally, recent sentinel surveillance showed substantial regional variation, including declines in coverage in some settings during the coronavirus disease 19 (COVID-19) pandemic [36]. Therefore, the patterns observed in Manhiça may not fully reflect other regions of the country where vaccine delivery and access are constrained. Additionally, other temporal and population-level factors such as indirect protection and improvements in WASH conditions may also have contributed to the observed declines [21–23]. The incidence of LSD was approximately 20 times higher than that of MSD, with an overall annual increase across all age groups. This may be aligned with reports indicating that rotavirus vaccines are more effective against severe than milder forms of the disease [24]. Nevertheless, the increasing frequency of other enteric pathogens may play a role in the continued or rising incidence of LDS, even with reduction of severe cases post-vaccine introduction. On the other hand, the high burden of LSD, particularly among children under two years of age, remains a public health concern due to its association with growth faltering, developmental impairment, and increased vulnerability to subsequent severe illness [25]. When comparing our findings to previous data from the GEMS (pre-vaccine) [7, 26], we observe a substantial reduction in rotavirus-attributable diarrhoea (19.15 and 5.63 percentage points for MSD and LSD, respectively) in infants post-rotavirus vaccine introduction. This is consistent with findings from the Global Paediatric Diarrhoea Surveillance network and other studies conducted post-vaccine introduction, which also reported persistent dominance of rotavirus among diarrhoeal pathogens despite vaccine rollout [10, 27]. These observations underscore the need for further research to evaluate the factors that may influence rotavirus vaccine performance in LMICs, including malnutrition, intestinal microbiota, interference of maternal antibodies, and environmental enteric dysfunction [28].

EAEC had the highest AF among MSD cases aged 12–23 months, surpassing both rotavirus and adenovirus 40/41. This finding contrasts with previous reports, where EAEC was not significantly associated with diarrhoea neither before nor after rotavirus vaccine introduction, including reports from Mozambique, where ETEC-ST rather than EAEC was associated with diarrhoea in children aged 12–23 months [26, 27]. Moreover, the pathogenic role of EAEC remains debated, as some studies suggest that it is primarily associated with subclinical infections, or that its association with diarrhoea depends on the presence of specific virulence factors [29, 30]. These discrepancies highlight the need for further molecular characterization of EAEC isolates to clarify their virulence profiles and role in disease induction. In our study, adenovirus 40/41, a well-established diarrhoeal pathogen, was shown to be responsible for cases of diarrhoea and has gained increased recognition post-rotavirus vaccine introduction. Additionally, studies from LMICs have reported that other viruses, such as adenovirus, norovirus, and sapovirus, may occupy the ecological niche left vacant by rotavirus post-vaccine introduction [31, 32].

In our study, *Cryptosporidium* spp. had an AF only for LSD among children under two years of age, and the estimated fraction surpassed that of rotavirus among children aged 12–23 months. While this may suggest a changing pattern of infection in post-rotavirus vaccine introduction, caution is warranted in interpretation, as *Cryptosporidium* spp. has been recognized as an important cause of severe diarrhoea both pre- and post-vaccine introduction [9, 26]. Recent molecular epidemiological evidence from Mozambique further supports the importance of *Cryptosporidium*, with *C. hominis* identified as the predominant species and the anthroponotic Ib family, particularly the IbA9G3 subtype, being the most frequently detected subtype [33]. The persistence of LSD and the substantial contribution of *Cryptosporidium* among young children may therefore “unmask” an important component of the diarrhoeal disease burden that may receive less attention when surveillance focuses primarily on severe disease. Given the association of recurrent or persistent diarrhoeal disease with growth faltering and impaired child development, these findings underscore the need for further investigation of *Cryptosporidium* species and subtypes, transmission pathways, and pathogenicity, as well as its potential contribution to longer-term health outcomes. Nevertheless, factors such as gradual improvements in WASH conditions, as well as enhanced HIV management and prevention of mother-to-child transmission, may help explain our findings.

The findings we report in this study should be interpreted considering some limitations. First, MSD recruitment started earlier than recruitment of LSD cases and community controls, introducing potential temporal differences between the groups. Calendar period was not explicitly accounted for in the pathogen-specific AF models, and the analysis did not incorporate the original matching structure. Changes over time in pathogen circulation, rotavirus vaccine coverage, or other epidemiological and healthcare factors may therefore have influenced case–control comparisons. The AF should consequently be interpreted as crude, model-based pathogen-specific estimates from the available case–control sample, and comparisons across groups and calendar periods should be interpreted with caution. Second, the only bacterial pathogen isolated in this study was *E. coli*. This limited detection may reflect the lower sensitivity of the conventional bacteriological methods employed, particularly when compared with more sensitive diagnostic techniques, such as quantitative molecular methods [10, 11, 34].

## Conclusions

Following the introduction and scale-up of rotavirus vaccination in Manhiça, MSD incidence declined among younger children. LSD incidence remained high, underscoring the continued burden of milder diarrhoea from other enteric pathogens. Rotavirus was the leading cause of both MSD and LSD in children aged 0–11 months. Other pathogens such as EAEC, adenovirus 40/41, sapovirus, and *Cryptosporidium* spp. were notably associated with diarrhoea (MSD and LSD). These findings reinforce the importance of continued surveillance and research into diarrhoea pathogens to inform future prevention strategies and guide vaccine policy.

## Supplementary Information

Supplementary material 1.

Supplementary material 2.

Supplementary material 3.

## Data Availability

All data generated or analysed during this study are included in this published article [and its supplementary information files].

## Abbreviations

MSD: Moderate to Severe Diarrhoea
LSD: Less Severe Diarrhoea
CYAR: Child-years at risk
GEMS: The Global Enteric Multicenter Study
AF: Attributable fraction
CISM: Centro de Investigação em Saúde de Manhiça
ETEC-ST: Heat-stable enterotoxin–producing enterotoxigenic *Escherichia coli*
HDSS: Health and Demographic Surveillance System
OPD: Outpatient visits
INPD: Inpatient visits
BGS: Buffered glycerol saline
ELISA: Enzyme-Linked Immunosorbent Assays
ETEC-LT: Heat-labile enterotoxin–producing enterotoxigenic *E. coli*
EPEC: Enteropathogenic *E. coli*
EAEC: Enteroaggregative *E. coli*
MCIR: Minimum community incidence rate
IRR: Incidence rate ratio
LMICs: Low and middle income countries
WASH: Water, Sanitation and Hygiene
WHO: World Health Organization

## References

[1] Mwenda JM, Mandomando I, Worwui AK, Gacic-Dobo M, Katsande R, Bwaka AM, et al. A decade of rotavirus vaccination in the World Health Organization African Region: an in-depth analysis of vaccine coverage from 2012 to 2023. Vaccine. 2012;48(Suppl C):126768. 10.1016/j.vaccine.2025.126768. 39890559

[2] Mutevedzi PC, Madewell ZJ, Kotloff KL, Bassat Q, Chirinda PJ, Cossa ACA, et al. Use of minimally invasive tissue sampling to determine the contribution of diarrheal diseases to under-five mortality and associated co-morbidities and co-infections in children with fatal diarrheal diseases in Africa and Bangladesh. PLoS Glob Public Health. 2025;5:e0004772. 10.1371/journal.pgph.0004772. PMC12193650 40561131

[3] WHO. Diarrhoeal disease. World Health Organization. 2024. https://www.who.int/news-room/fact-sheets/detail/diarrhoeal-disease. Accessed 15 Dec 2025.

[4] United Nations Children’s Fund (UNICEF). One is too many: ending child deaths from pneumonia and diarrhoea. UNICEF. 2016. https://data.unicef.org/resources/one-many-ending-child-deaths-pneumonia-diarrhoea/. Accessed 15 Dec 2025.

[5] WHO. Meeting of the immunization Strategic Advisory Group of Experts, April 2009–conclusions and recommendations. Wkly Epidemiol Rec. 2009;84:220–36. 19499606

[6] Kotloff KL, Nataro JP, Blackwelder WC, Nasrin D, Farag TH, Panchalingam S, et al. Burden and aetiology of diarrhoeal disease in infants and young children in developing countries (the Global Enteric Multicenter Study, GEMS): a prospective, case-control study. Lancet. 2013;382:209–22. 10.1016/S0140-6736(13)60844-2. 23680352

[7] Kotloff KL, Nasrin D, Blackwelder WC, Wu Y, Farag T, Panchalingham S, et al. The incidence, aetiology, and adverse clinical consequences of less severe diarrhoeal episodes among infants and children residing in low-income and middle-income countries: a 12-month case-control study as a follow-on to the Global Enteric Multicenter Study (GEMS). Lancet Glob Health. 2019;7:e568–84. 10.1016/S2214-109X(19)30076-2. PMC6484777 31000128

[8] Manjate F, Quintó L, Chirinda P, Acácio S, Garrine M, Vubil D, et al. Impact of rotavirus vaccination on diarrheal hospitalizations in children younger than 5 years of age in a rural southern Mozambique. Vaccine. 2022;40:6422–30. 10.1016/j.vaccine.2022.09.050. PMC9589241 36192272

[9] Buchwald AG, Verani JR, Keita AM, Jahangir Hossain M, Roose A, Sow SO, et al. Etiology, presentation, and risk factors for diarrheal syndromes in 3 Sub-Saharan African countries after the introduction of rotavirus vaccines from the Vaccine Impact on Diarrhea in Africa (VIDA) Study. Clin Infect Dis. 2023;76(Supplement_1):S12-22. 10.1093/cid/ciad022. PMC10116565 37074436

[10] Cohen AL, Platts-Mills JA, Nakamura T, Operario DJ, Antoni S, Mwenda JM, et al. Aetiology and incidence of diarrhoea requiring hospitalisation in children under 5 years of age in 28 low-income and middle-income countries: findings from the Global Pediatric Diarrhea Surveillance network. BMJ Glob Health. 2022;7:e009548. 10.1136/bmjgh-2022-009548. PMC9445824 36660904

[11] Operario DJ, Platts-Mills JA, Nadan S, Page N, Seheri M, Mphahlele J, et al. Etiology of severe acute watery diarrhea in children in the global rotavirus surveillance network using quantitative polymerase chain reaction. J Infect Dis. 2017;216:220–7. 10.1093/infdis/jix294. PMC5853801 28838152

[12] Manjate F, João ED, Chirinda P, Garrine M, Vubil D, Nobela N, et al. Molecular epidemiology of rotavirus strains in symptomatic and asymptomatic children in Manhiça District, Southern Mozambique 2008–2019. Viruses. 2008;14:134. 10.3390/v14010134. PMC8781303 35062336

[13] Munlela B, João ED, Donato CM, Strydom A, Boene SS, Chissaque A, et al. Whole genome characterization and evolutionary analysis of G1P[8] rotavirus A strains during the pre- and post-vaccine periods in Mozambique (2012–2017). Pathogens. 2012;9:1026. 10.3390/pathogens9121026. PMC7762294 33291333

[14] Chirinda P, Manjate F, Garrine M, Messa A, Nobela N, Vubil D, et al. Detection of enteric viruses in children under five years of age before and after rotavirus vaccine introduction in Manhiça District, Southern Mozambique, 2008–2019. Viruses. 2008;16:1159. 10.3390/v16071159. PMC11281453 39066321

[15] Nhambirre OL, Cossa-Moiane I, Bauhofer AFL, Chissaque A, Lobo ML, Matos O, et al. Intestinal parasites in children up to 14 years old hospitalized with diarrhea in Mozambique, 2014–2019. Pathogens. 2014;11:353. 10.3390/pathogens11030353. PMC8954659 35335676

[16] Sacoor C, Nhacolo A, Nhalungo D, Aponte JJ, Bassat Q, Augusto O, et al. Profile: Manhica Health Research Centre (Manhica HDSS). Int J Epidemiol. 2013;42:1309–18. 10.1093/ije/dyt148. 24159076

[17] Nhacolo A, Jamisse E, Augusto O, Matsena T, Hunguana A, Mandomando I, et al. Cohort profile update: Manhiça Health and Demographic Surveillance System (HDSS) of the Manhiça Health Research Centre (CISM). Int J Epidemiol. 2021;50:395–402. 10.1093/ije/dyaa218. PMC8128467 33452521

[18] Panchalingam S, Antonio M, Hossain A, Mandomando I, Ochieng B, Oundo J, et al. Diagnostic microbiologic methods in the GEMS-1 case/control study. Clin Infect Dis. 2012;55(suppl_4):S294-302. 10.1093/cid/cis754. PMC3502308 23169941

[19] Gupta A, Sarker G, Rout A, Mondal T, Pal R. Risk correlates of diarrhea in children under 5 years of age in slums of Bankura, West Bengal. J Glob Infect Dis. 2015;7:23. 10.4103/0974-777X.150887. PMC4338445 25722616

[20] Chavers T, Cates J, Burnett E, Parashar UD, Tate JE. Indirect protection from rotavirus vaccines: a systematic review. Expert Rev Vaccines. 2024;23:789–95. 10.1080/14760584.2024.2395534. PMC12107528 39167375

[21] Ngabo F, Tate JE, Gatera M, Rugambwa C, Donnen P, Lepage P, et al. Effect of pentavalent rotavirus vaccine introduction on hospital admissions for diarrhoea and rotavirus in children in Rwanda: a time-series analysis. Lancet Glob Health. 2016;4:e129–36. 10.1016/S2214-109X(15)00270-3. PMC11976669 26823214

[22] Merid MW, Alem AZ, Chilot D, Belay DG, Kibret AA, Asratie MH, et al. Impact of access to improved water and sanitation on diarrhea reduction among rural under-five children in low and middle-income countries: a propensity score matched analysis. Trop Med Health. 2023;51:36. 10.1186/s41182-023-00525-9. PMC10268525 37322559

[23] Wolf J, Hubbard S, Brauer M, Ambelu A, Arnold BF, Bain R, et al. Effectiveness of interventions to improve drinking water, sanitation, and handwashing with soap on risk of diarrhoeal disease in children in low-income and middle-income settings: a systematic review and meta-analysis. Lancet. 2022;400:48–59. 10.1016/S0140-6736(22)00937-0. PMC9251635 35780792

[24] Tapia MD, Armah G, Breiman RF, Dallas MJ, Lewis KDC, Sow SO, et al. Secondary efficacy endpoints of the pentavalent rotavirus vaccine against gastroenteritis in sub-Saharan Africa. Vaccine. 2012;30:A79-85. 10.1016/j.vaccine.2012.01.022. 22520141

[25] Assis AMO, Barreto ML, Santos LMP, Fiaccone R, Da Silva Gomes GS. Growth faltering in childhood related to diarrhea: a longitudinal community based study. Eur J Clin Nutr. 2005;59:1317–23. 10.1038/sj.ejcn.1602245. 16077743

[26] Nhampossa T, Mandomando I, Acacio S, Quintó L, Vubil D, Ruiz J, et al. Diarrheal disease in rural Mozambique: burden, risk factors and etiology of diarrheal disease among children aged 0–59 months seeking care at health facilities. PLoS ONE. 2015;10:e0119824. 10.1371/journal.pone.0119824. PMC4431848 25973880

[27] Iturriza-Gómara M, Jere KC, Hungerford D, Bar-Zeev N, Shioda K, Kanjerwa O, et al. Etiology of diarrhea among hospitalized children in Blantyre, Malawi, following rotavirus vaccine introduction: a case-control study. J Infect Dis. 2019;220:213–8. 10.1093/infdis/jiz084. PMC6581894 30816414

[28] Parker EPK, Bronowski C, Sindhu KNC, Babji S, Benny B, Carmona-Vicente N, et al. Impact of maternal antibodies and microbiota development on the immunogenicity of oral rotavirus vaccine in African, Indian, and European infants. Nat Commun. 2021;12:7288. 10.1038/s41467-021-27074-1. PMC8674366 34911947

[29] Durand D, Contreras CA, Mosquito S, Ruíz J, Cleary TG, Ochoa TJ. Pic gene of enteroaggregative *Escherichia coli* and its association with diarrhea in Peruvian children. Pathog Dis. 2016;74:ftw054. 10.1093/femspd/ftw054. PMC5985504 27307104

[30] Lima AAM, Soares AM, Filho JQS, Havt A, Lima IFN, Lima NL, et al. Enteroaggregative *Escherichia coli* subclinical infection and coinfections and impaired child growth in the MAL‐ED cohort study. J Pediatr Gastroenterol Nutr. 2018;66:325–33. 10.1097/mpg.0000000000001717. 29356769

[31] Varghese T, Mills JAP, Revathi R, Antoni S, Soeters HM, Emmanuel Njambe TO, et al. Etiology of diarrheal hospitalizations following rotavirus vaccine implementation and association of enteric pathogens with malnutrition among under-five children in India. Gut Pathog. 2024;16:22. 10.1186/s13099-024-00599-8. PMC11005126 38600552

[32] Ballard S-B, Requena D, Mayta H, Sanchez GJ, Oyola-Lozada MG, Colquechagua Aliaga FD, et al. Enteropathogen changes after rotavirus vaccine scale-up. Pediatrics. 2022;149:e2020049884. 10.1542/peds.2020-049884. PMC9647525 34918158

[33] Nhambirre O, Lobo ML, Cossa-Moiane I, Bauhofer A, Deus ND, Matos O. Risk factors and circulating subtypes of *Cryptosporidium* spp. and *Giardia duodenalis* in hospitalized children in Mozambique. Microorganisms. 2025;13:196. 10.3390/microorganisms13010196. PMC11767712 39858964

[34] Liu J, Platts-Mills JA, Juma J, Kabir F, Nkeze J, Okoi C, et al. Use of quantitative molecular diagnostic methods to identify causes of diarrhoea in children: a reanalysis of the GEMS case-control study. Lancet. 2016;388:1291–301. 10.1016/S0140-6736(16)31529-X. PMC5471845 27673470

[35] Newson, RB. Attributable and Unattributable Risks and Fractions and other Scenario Comparisons. Stata J. 2013;13(4):672-698. 10.1177/1536867X1301300402.

[36] Cassocera M, Bauhofer AFL, Chissaque A, Munlela B, Guimarães E, Isaías T, et al. Regional difference on rotavirus vaccine coverage in children with diarrhea in Mozambique, before and during COVID-19 pandemic: a cross-sectional analysis. BMC Infect Dis. 2025;25:382. 10.1186/s12879-025-10750-8. PMC11924852 40108531

