# Supplementary material for: Incidence and aetiology of diarrhoeal disease among children under five years in the post-rotavirus vaccine introduction era in Manhiça District, Mozambique
Source: Trop Med Health. 2026 Oct 19;54:223. doi: 10.1186/s41182-026-01095-2 (PMC13679116; doi:10.1186/s41182-026-01095-2)
Supplement: Supplementary file 1 [file 41182_2026_1095_MOESM1_ESM.docx]

**Table S1.** List of Primer sequences and amplicon sizes used in the RT-PCR multiplex to detect norovirus GI and GII, sapovirus and astrovirus

| Virus | Primer | Primer sequence 5´-3´ | Amplicon bp |
| --- | --- | --- | --- |
| Norovirus GI | G1SKR | CCAACCCARCCATTRTACA | 330 |
|  | G1SKF | CTGCCCGAATTYGTAAATGA |  |
| Norovirus GII | G2SKR | CCRCCNGCATRHCCRTTRTACAT | 387 |
|  | COG2F | CARGARBCNATGTTYAGRTGGATGAG |  |
| Sapovirus | SLV5749 | CGGRCYTCAAAVSTACCBCCCCA | 434 |
|  | SLV5317 | CTCGCCACCTACRAWGCBTGGTT |  |
| Astrovirus | 82b | GTGAGCCACCAGCCATCCCT | 719 |
|  | PreCAP1 | GGACTGCAAAGCAGCTTCGTG |  |

Reference

Panchalingam S, Antonio M, Hossain A, Mandomando I, Ochieng B, Oundo J, et al. Diagnostic Microbiologic Methods in the GEMS-1 Case/Control Study. Clinical Infectious Diseases 2012;55:S294–302. https://doi.org/10.1093/cid/cis754.
