# Supplementary material for: Incidence and aetiology of diarrhoeal disease among children under five years in the post-rotavirus vaccine introduction era in Manhiça District, Mozambique
Source: Trop Med Health. 2026 Oct 19;54:223. doi: 10.1186/s41182-026-01095-2 (PMC13679116; doi:10.1186/s41182-026-01095-2)
Supplement: Supplementary file 2 [file 41182_2026_1095_MOESM2_ESM.docx]

**Table S2.** List of Primer sequences and amplicon sizes used in the RT-PCR multiplex to detect Diarrheagenic *E.coli* pathotypes

| Pathotype | Primer | Target gene | Primer sequence 5´-3´ | Amplicon bp |
| --- | --- | --- | --- | --- |
| Enterotoxigenic *E. coli* (ETEC) | LT-F | elt | CACACGGAGCTCCTCAGTC | 508 |
|  | LT-R |  | CCCCCAGCCTAGCTTAGTTT |  |
|  | ST-F | est | GCTAAACCAGTAG/AGGTCTTCAAAA | 147 |
|  | ST-R |  | CCCGGTACAG/AGCAGGATTACAACA |  |
| Enteropatogenic *E. coli* (EPEC) | BFPA-F | bfpA | GGAAGTCAAATTCATGGGGG | 367 |
|  | BFPA-R |  | GGAATCAGACGCAGACTGGT |  |
|  | EAE-F | eae | CCCGAATTCGGCACAAGCATAAGC | 881 |
|  | EAE-R |  | CCCGGATCCGTCTCGCCAGTATTCG |  |
| Enteroaggregative *E. coli* (EAEC) | CVD432F | aatA | CTGGCGAAAGACTGTATCAT | 630 |
|  | CVD432R |  | CAATGTATAGAAATCCGCTGTT |  |
|  | AAIC F | aaiC | ATTGTCCTCAGGCATTTCAC | 215 |
|  | AAIC R |  | ACGACACCCCTGATAAACAA |  |

**Reference**

Panchalingam S, Antonio M, Hossain A, Mandomando I, Ochieng B, Oundo J, et al. Diagnostic Microbiologic Methods in the GEMS-1 Case/Control Study. Clinical Infectious Diseases 2012;55:S294–302. https://doi.org/10.1093/cid/cis754.
