# Supplementary material for: Incidence and aetiology of diarrhoeal disease among children under five years in the post-rotavirus vaccine introduction era in Manhiça District, Mozambique
Source: Trop Med Health. 2026 Oct 19;54:223. doi: 10.1186/s41182-026-01095-2 (PMC13679116; doi:10.1186/s41182-026-01095-2)
Supplement: Supplementary file 3 [file 41182_2026_1095_MOESM3_ESM.docx]

**Table S3.** Detection of enteric pathogens in Moderate to severe (MSD) and Less severe (LSD) diarrhoea cases and controls, according to the age strata in Manhiça District, Mozambique, from 2015-2021.

| Pathogen | Overal | 0-11 months | | | 12-23 months | | | 24-59 months | | |
| --- | --- | --- | --- | --- | --- | --- | --- | --- | --- | --- |
|  | **% (n/N)** | **MSD cases % (n/N)** | **LSD % (n/N)** | **Controls % (n/N)** | **MSD cases % (n/N)** | **LSD % (n/N)** | **Controls % (n/N)** | **MSD cases % (n/N)** | **LSD % (n/N)** | **Controls % (n/N)** |
| **Protozoa** |  |  |  |  |  |  |  |  |  |  |
| *G. duodenalis* | 28 (488/1762) | 5 (7 / 149) | 11 (21/192) | 15 (60/411) | 23 (23/99) | 37 (48/130) | 39 (170/439) | 29 (15/51) | 44 (30/68) | 51 (114/223) |
| *E. histolytica* | 11 (199/1751) | 12 (18/148) | 8 (16/192) | 10 (39/407) | 8 (8/97) | 17 (22/126) | 11 (48/439) | 19 (10/52) | 13 (9/67) | 13 (29/223) |
| *Cryptosporidium spp.* | 4 (75/1772) | 9 (14/150) | 8 (15/193) | 3 (14/414) | 6 (6/97) | 8 (10/129) | 2 (11/441) | 0 (0/52) | 3 (2/70) | 1 (3/226) |
| **Viruses** |  |  |  |  |  |  |  |  |  |  |
| Rotavirus | 17 (307/1777) | 28 (43/151) | 21 (41/191) | 10 (42/416) | 26 (26/99) | 20 (26/131) | 14 (61/441) | 25 (13/52) | 26 (18/70) | 16 (37/226) |
| Norovirus | 7 (68/971) | 10 (12/123) | 12 (16/132) | 6 (11/195) | 8 (6/73) | 3 (3/88) | 6 (13/207) | 0 (0/29) | 15 (7/47) | 0 (0/77) |
| Adenovirus 40/41 | 2 (32/1763) | 2 (3/145) | 2 (3/191) | 1 (3/412) | 14 (14/99) | 3 (4/130) | 1 (3/439) | 0 (0/52) | 3 (2/69) | 0 (0/226) |
| Astrovirus | 6 (55/971) | 6 (7/123) | 6 (8/132) | 3 (6/195) | 3 (2/73) | 8 (7/88) | 7 (15/208) | 10 (3/29) | 9 (4/47) | 4 (3/76) |
| Sapovirus | 3 (32/969) | 2 (2/123) | 8 (11/131) | 2 (3/194) | 1 (1/73) | 6 (5/88) | 3 (6/207) | 0 (0/29) | 9 (4/47) | 0 (0/77) |
| **Bacteria** |  |  |  |  |  |  |  |  |  |  |
| ETEC ST | 4 (46/1107) | 10 (10/105) | 3 (4/121) | 2 (6/265) | 9 (6/67) | 6 (5/87) | 3 (9/264) | 7 (2/28) | 4 (2/50) | 2 (2/120) |
| ETEC LT | 4 (40/1107) | 1 (1/105) | 3 (4/121) | 3 (9/265) | 4 (3/67) | 2 (2/87) | 5 (13/264) | 11 (3/28) | 4 (2/50) | 3 (3/120) |
| EPEC typical | 7 (75/1107) | 10 (11/105) | 3 (4/121) | 9 (23/265) | 10 (7/67) | 7 (6/87) | 8 (21/264) | 4 (1/28) | 0 (0/50) | 2 (2/120) |
| EPEC atypical | 1 (16/1107) | 1 (1/105) | 3 (4/121) | 1 (3/265) | 1 (1/67) | 1 (1/87) | 2 (4/264) | 4 (1/28) | 0 (0/50) | 1 (1/120) |
| EAEC (*aatA/aaiC*) | 18 (194/1107) | 27 (28/105) | 26 (32/121) | 19 (51/265) | 24 (16/67) | 16 (14/87) | 13 (33/264) | 18 (5/28) | 14 (7/50) | 7 (8/120) |

*Abbreviations:* ETEC ST- Enterotoxigenic *E. coli* producing heat-stable enterotoxin; ETEC LT - Enterotoxigenic *E. coli* producing heat-labile enterotoxin; EPEC- Enteropathogenic *E. coli*; EAEC - Enteroaggregative *E. coli.*
